# Efficacy and safety of pulsed radiofrequency as a method of dorsal root ganglia stimulation for treatment of non-neuropathic pain: a systematic review

**DOI:** 10.1186/s12871-020-01023-9

**Published:** 2020-05-04

**Authors:** Ivana Vuka, Svjetlana Došenović, Tihana Marciuš, Lejla Ferhatović Hamzić, Katarina Vučić, Damir Sapunar, Livia Puljak

**Affiliations:** 1 Laboratory for Pain Research, University of Split School of Medicine, Šoltanska 2, 21000 Split, Croatia; 2 Department of Anesthesiology, Reanimatology and Intensive Care, University Hospital Split, Spinčićeva 1, 21000 Split, Croatia; 3 Center for Translational and Clinical Research, Department of Proteomics, University of Zagreb School of Medicine, Šalata 3, 10000 Zagreb, Croatia; 4 Department for Safety and Efficacy Assessment of Medicinal Products, Agency for Medicinal Products and Medical Devices, Ksaverska cesta 4, 10000 Zagreb, Croatia; 5 Center for Evidence-Based Medicine and Health Care, Catholic University of Croatia, Ilica 242, 10000 Zagreb, Croatia

**Keywords:** Chronic pain, Non-neuropathic pain, Pulsed radiofrequency, Dorsal root ganglion

## Abstract

**Background:**

We systematically reviewed the evidence on the efficacy and safety of dorsal root ganglion (DRG) targeted pulsed radiofrequency (PRF) versus any comparator for treatment of non-neuropathic pain.

**Methods:**

We searched MEDLINE, CINAHL, Embase, PsycINFO, clinicaltrials.gov and WHO clinical trial register until January 8, 2019. All study designs were eligible. Two authors independently conducted literature screening. Primary outcomes were pain intensity and serious adverse events (SAEs). Secondary outcomes were any other pain-related outcome and any other safety outcome that was reported. We assessed the risk of bias using the Cochrane tool and Risk of Bias In Non-randomized Studies of Interventions (ROBINS-I). We conducted narrative evidence synthesis and assessed the conclusiveness of included studies regarding efficacy and safety.

**Results:**

We included 17 studies with 599 participants, which analyzed various pain syndromes. Two studies were randomized controlled trials; both included participants with low back pain (LBP). Non-randomized studies included patients with the following indications: LBP, postsurgical pain, pain associated with herpes zoster, cervicogenic headache, complex regional pain syndrome type 1, intractable vertebral metastatic pain, chronic scrotal and inguinal pain, occipital radiating pain in rheumatoid arthritis and chronic migraine. In these studies, the PRF was usually initiated after other treatments have failed. Eleven studies had positive conclusive statements (11/17) about efficacy; the remaining had positive inconclusive statements. Only three studies provided conclusiveness of evidence statements regarding safety – two indicated that the evidence was positive conclusive, and one positive inconclusive. The risk of bias was predominantly unclear in randomized and serious in non-randomized studies.

**Conclusion:**

Poor quality and few participants characterize evidence about benefits and harms of DRG PRF in patients with non-neuropathic pain. Results from available studies should only be considered preliminary. Not all studies have reported data regarding the safety of the intervention, but those that did, indicate that the intervention is relatively safe. As the procedure is non-destructive and early results are promising, further comparative studies about PRF in non-neuropathic pain syndromes would be welcomed.

## Background

Chronic pain is one of the major public health issues worldwide and is one of the leading causes of years lived with disability [1]. Estimates on the prevalence of chronic pain in the general population vary, ranging from 11% [2] up to 64% [3]. These different estimates are mostly due to differences in the definition of chronic pain regarding the duration of symptoms (3 vs. 6 months) and the wording of questions used for assessing chronic pain [4]. Besides its major clinical impact and costs for the healthcare system, chronic pain impairs patients’ quality of life, as well as their ability to work and function, causing massive indirect socioeconomic costs worldwide [5]. Chronic pain asserts this major impact on individuals, health systems and society because of inadequate treatment modalities.

Pulsed radiofrequency (PRF) emerged as a therapeutic treatment for various painful conditions, including both neuropathic and non-neuropathic pain [6–8]. PRF has been described as “a non-neurodestructive therapy in pain management ”[9]. PRF is a minimally invasive intervention, which involves the application of pulses of electric current, created at the tip of an electrode, without a harmful increase in the temperature [9].

It has been suggested that a dorsal root ganglion (DRG) is a desirable target for the treatment of pain [10]. PRF application close to dorsal root could alleviate neuropathic pain [11]. However, we have observed an increasing number of studies on chronic pain, reporting use of DRG targeted PRF treatment of non-neuropathic pain in humans. Therefore, we aimed to conduct a systematic review about the evidence on the efficacy and safety of DRG targeted PRF treatment of non-neuropathic pain.

## Methods

### Study design

We published a systematic review protocol a priori in the PROSPERO database (registration number: CRD42017076502). Since the original protocol covered extremely wide scope and heterogeneous interventions, subsequently we divided the original protocol into a separate assessment of DRG targeted electrical field stimulation (EFS) [12] and PRF. The systematic review was performed following the PRISMA statement and Center for Reviews and Dissemination (CRD) manuals.

### Eligibility criteria

#### Participants, intervention and study designs

We included primary studies with participants suffering from various painful conditions which are not currently classified as purely of neuropathic origin (i.e. non-neuropathic pain). In case that condition was defined of both origins, neuropathic and non-neuropathic, such as post-surgical pain or low back pain we included such condition. We excluded studies where PRF treatment was used for neuropathic pain as it is defined in the guidelines of the International Association for the Study of Pain (IASP). We used the IASP classification of chronic pain for ICD-11. We chose to include both randomized controlled trials (RCTs) as well as non-randomized study designs (NRSDs) because we expected a few RCTs in this research area, and we wanted to provide a comprehensive picture of evidence in this field of research. We used Cochrane Handbook for Reviews of Interventions to define the design of included studies. Manuscripts that included more than 10 participants were classified as case series, while those that included less than 10 participants were defined as case reports [13]. We only included studies where PRF treatment was directed to the DRG, including a combination of PRF with other therapies. If the study only reported results about efficacy, and safety was not reported, we still included such a study to get comprehensive evidence synthesis regarding efficacy.

#### Outcome measures

Primary outcomes were: pain intensity and serious adverse events (SAEs). For primary outcome, we reported any outcome measures, as reported in included manuscripts. Secondary outcomes for efficacy were any other pain-related outcomes, and for safety any other safety data, including non-serious adverse events and other complications regarding tested intervention.

### Search strategy and information source

We searched four databases: MEDLINE via Ovid, Embase via Ovid, CINAHL and PsycINFO via EBSCOhost (Supplementary Table 1). Databases were searched from the date of inception until January 8, 2019 with no restriction regarding the language. Records were then exported to the EndNote X5 citation software (Clarivate Analytics, Boston, MA, USA) and duplicates removed. Furthermore, reference lists of all included studies and their citations were downloaded from Web of Science and screened to find additional eligible studies. ClinicalTrials.gov and World Health Organization’s International Clinical Trial Registry Platform (WHO ICTRP) were searched to identify ongoing studies.

### Study selection

Reviewers independently screened each title/abstract of retrieved records as well as full-texts of retrieved studies for possible inclusion (authors LFH, IV, TM and SD participated in screening). Discrepancies were resolved by another author (DS).

### Data extraction

Independent data extraction was performed by two authors for each data point (authors: IV, and TM or KV). We extracted the following data: the surname of the first author, year of publication, study design, details about intervention (treatment protocol and device used), comparator, inclusion and exclusion criteria, number of participants, baseline characteristics of participants, follow-up period, DRG level treated and outcomes about efficacy and safety.

### Risk of bias assessment

We used the Cochrane Risk of Bias (RoB) tool (version from 2011) to assess RoB in RCTs and the Risk of Bias In Non-randomized Studies of Interventions (ROBINS-I) tool for cohort type studies. RoB was analyzed independently by two authors (IV, and SD or KV). Discrepancies were resolved by another author (LP).

### Synthesis of results

Due to the heterogeneity of included studies, it was not possible to conduct a meta-analysis, even though we have planned to do it in our study protocol. For this reason, we conducted a narrative and tabular synthesis of results. We also conducted an analysis of conclusiveness about efficacy and safety of the treatment in the abstracts of included studies. We divided conclusiveness statements into five categories: positive conclusive (favorable conclusion in favor of PRF), positive inconclusive (favorable conclusion, but with a note about insufficient or low quality evidence), negative conclusive (PRF not beneficial), negative inconclusive (PRF not beneficial, but with a note about insufficient or low quality evidence) and not reported.

## Results

**Fig. 1:**
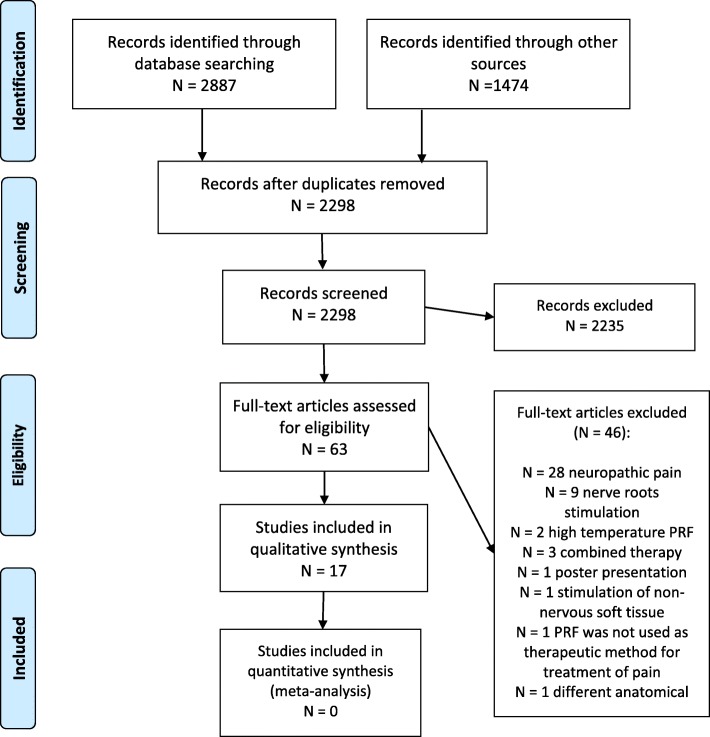
Flow chart of study inclusion

The flow chart in Fig. 1 shows the number of records analyzed in each screening phase. We screened 63 manuscripts in full text, and we finally included 17 manuscripts in this systematic review. Excluded studies, and reasons for their exclusion, are listed in Supplementary Table 2. The characteristics of the included studies are detailed in Table 1.

**Table 1:** Characteristics about efficacy and safety of included studies

Author and year/Study design (Cochrane handbook and study authors)	Interventions prior to PRF treatment	Number of participants (for each pain condition treated)	Follow-up	Outcome measures	Results: efficacy for pain intensity	Conclusion statement about efficacy	Results: serious adverse events	Results: any other safety data	Conclusion statement about safety
**Low back pain (LBP)**									
Holanda 2016 [14] RCT / pilot study	No other intervention	PRF treatment group *n* = 11; lidocaine injection group *n* = 7; laser irradiation treatment group *n* = 10	5 min and 1 month	1. Lumbar pain intensity by VAS presented as percentage of relative difference 2. Chronic LBP relief by PRS	**PRF group:** VAS relative difference at 5 min: 62.5% VAS relative difference at 1 month: 20% **Lidocaine injection group:** VAS relative difference at 5 min: 100% VAS relative difference at 1 month: 62.5% **Laser treatment group:** VAS relative difference at 5 min: 100% VAS relative difference at 1 month: 55%	Positive conclusive	No SAEs occurred	Some patients experienced only mild discomfort during procedure	Not reported
Lee 2018 [15] RCT / randomized, prospective, and comparative study	Diagnostic block + PRF group received diagnostic block with 1 ml of 2% bupivacaine and 1 ml of 2% triamcinolone.	Diagnostic block + PRF group (*n* = 30); PRF group (*n* = 30)	2 weeks, 1, 3 and 6 months	1. Pain intensity by NRS 2. Functional disabilities by ODI	**Diagnostic block + PRF group:** Baseline NRS: 8 (range 5–9); NRS at 2 weeks: 2 (range 1–7); NRS at 1 month: 2 (range 1–8); NRS at 3 months: 3 (range 1–8); NRS at 6 months: 4 (range 1–8). **PRF alone group:** Baseline NRS: 7.5 range (3–10); NRS at 2 weeks: 2 (range 1–9); NRS at 1 month: 2 (range 1–9); NRS at 3 months: 3 (range 1–9); NRS at 6 months: 4 (range 1–9). *P* values comparison between groups: NRS at 2 weeks: *P* = 0.302 NRS at 1 month: *P* = 0.690 NRS at 3 moths: *P* = 0.957 NRS at 6 months: *P* = 0.673	Positive inconclusive	Not reported	Not reported	Not reported
Yang 2010 [16] RCS / in vivo clinical trial	No other intervention	PaMNI system *n* = 16; conventional fluoroscopy *n* = 13	1 month	1. Pain intensity by VAS	**PaMNI group:** Baseline VAS: 5.8 (±2.3); VAS at 1 month: 4.1 (±2.1). *P* = 0.005. **Fluoroscopy group:** Baseline VAS: 6.5 (±2.2); VAS at 1 month: 5.3 (±2.8). *P* = 0.067. No statistical difference between groups at 1 month (*P* = 0.238).	Positive conclusive	Not reported	Not reported	Not reported
Hsu 2017 [17] BA / retrospective study	No other intervention	84	1 week after the treatment and at 3, 6, 9, 12 months and yearly postoperatively (for 3 years in total)	1. Pain intensity by VAS 2. Functional disabilities by ODI	Analysis of VAS scores for pain indicated significant reductions of low back pain during the 3-year follow-up for patients with all 4 types of lumbar lordosis.	Positive conclusive	No SAEs occurred	Cerebral spinal fluid leaking from the cannulas of two patients while the needle was being directed toward the DRG. This leakage ceased immediately after adjusting the location of the needle tip.	Specific adverse events mention, no overall conclusion about safety
Tsou 2010 [18] BA / not stated	No other intervention	Group A (CLBP without lower-limb pain) *n* = 49; Group B (CLBP with lower-limb pain) *n* = 78	From 1 week up to 3 years post-operatively	1. Pain intensity by VAS 2. Adverse events	**Group A, L2 treatment:** ≥50% VAS improvement: at 1 week: 25/49 (51.02%); at 3 months: 27/49 (55.1%); at 1 year 20/45 (44.44%). **Group B, L2 treatment:** ≥50% VAS improvement: at 1 week: 34/78 (43.59%); at 3 months: 37/78 (47.44%); at 1 year: 34/74 (45.95%).	Positive conclusive	No SAEs occurred	No obvious complications were observed	Positive conclusive
**Postsurgical pain**									
Albayrak 2017 [19] PCS / retrospective study of prospectively collected data	In the PRF group participants received prognostic diagnostic block prior to involvement.	PRF group (TENS + exercise + PRF) *n* = 22; TENS group (TENS + exercise) *n* = 17	15 days and 1-month post treatment and following last control examination. The mean follow-up time was 253.8 ± 109 days; for TENS group: 217 ± 114 days and for PRF group: 282.2 ± 97 days	1. Pain intensity by VAS 2. Degree of neuropathic pain reduction by DN4 3. Change in knee flexion by ROM 4. Functional status by WOMAC 5. Patient satisfaction Success was defined as at least 50% reduction to the VAS (activity, rest, night)	**PRF group activity:** baseline VAS: 6.6 (±1.5); VAS at 15 days: 3 (±1.4); VAS at 1 month: 3.9 (±2); VAS at last control: 3.5 (±2.4). **PRF group rest:** baseline VAS: 4.3 (±1.7); VAS at 15 days: 1.8 (±0.9); VAS at 1 month: 2.6 (±1.5); VAS at last control: 2 (±1.6). **PRF group night:** baseline VAS: 3.8 (±2.2); VAS at 15 days: 1.5 (±1.1); VAS at 1 month: 2.1 (±1.4); VAS at last control: 1.7 (±1.4). **TENS group activity:** baseline VAS: 5.9 (±1.9); VAS at 15 days: 3.8 (±2.2); VAS at 1 month: 4.3 (±2.2); VAS at last control: 4.4 (±2.1). **TENS group rest:** baseline VAS: 5 (±2.4); VAS at 15 days: 2.6 (±2.4); VAS at 1 month: 3.4 (±2.5); VAS at last control: 2.8 (±2.1). **TENS group night:** baseline VAS: 4.3 (±2.6); VAS at 15 days: 2.1 (±2.7); VAS at 1 month: 2.7 (±2.6); VAS at last control: 2.6 (±1). Significant difference achieved in an improvement of at least 50% on the VAS scores at activity following the last control examination between the two groups (*P* = 0.006), but not on the VAS scores at rest and night (*P* > 0.05).	Positive conclusive	No SAEs occurred	No complications were observed	Not reported
Cohen 2006 [20] RCS / retrospective data analysis	No other intervention	PRF DRG group *n* = 13; PRF ICN group *n* = 15; MM group *n* = 21	6 weeks, 3 months	1. Pain intensity by VAS 2. Answers to 2 questions evaluating patient satisfaction and functional improvement Successful was defined as ≥50% pain reduction on VAS and affirmative answer to 2 questions	No separate VAS scores shown in manuscript, success was achieved as follows.: **PRF DRG group:** 6 weeks: 61.5% 3 months: 53.8% **PRF ICN group:** 6 weeks: 21.4% 3 months: 6.7% **MM group:** 6 weeks: 27.3% 3 months: 19.9% Effect did not reach statistical significance at 6 weeks (*P* = 0.12). At 3 months, success rate for PRF DRG group was significantly greater than for those patients treated with PRF ICN (*P* = 0.01), and approached significance when compared with MM (*P* = 0.06).	Positive conclusive	Small incidental pneumothorax was found during a routine scan of the lung fields after PRF DRG. This patient was not symptomatic and was treated conservatively with observation.	No other complications occurred	Not reported
Fam 2018 [21] BA / single arm intervention study	Steroid injections with 1 ml of bupivacaine 0.25% and 1 ml of dexamethasone 4 mg in a total volume of 2 ml immediately after PRF procedure.	*n* = 100	1 week, 1, 3 and 6 months	1. Pain intensity by VAS 2. Quality of life by QOLS 3. Change in use of pain medication 4. Adverse effects 5. Patient satisfaction	Baseline VAS: 7.48 ± 1.46 (median: 8); VAS at 1 week: 5.01 ± 2.61 (median: 5) (*P* = 0.032344); VAS at 1 month: 3.26 ± 2.37 (median: 3) (*P* < 0.0001); VAS at 3 months: 4.44 ± 2.8 (median: 4) (*P* = 0.00139); VAS at 6 months: 4.7 ± 2.88 (median: 4) (*P* = 0.0057).	Positive inconclusive	No SAEs occurred	Pain at the needling site, fever of unknown etiology at the night of intervention, mild to moderate elevation of glucose level in portion of diabetic participants	Positive inconclusive
**Pain associated with herpes zoster**									
Kim 2017 [6] RCS / retrospective cohort study	No other intervention	PRF group *n* = 20; continuous epidural group (ropivacaine) *n* = 22	1, 3 and 6 months	1. Pain intensity by NRS 2. Dose of anticonvulsants and analgesics	**PRF group:** baseline NRS: 6.30 ± 0.98 **Continuous epidural group:** baseline NRS: 6.73 ± 0.88 NRS values were significantly lower in PRF group from 1 to 3 months and 6 months after the procedure (*P* = 0.029) than those in continuous epidural group.	Positive conclusive	No SAEs occurred	1 patient complained of pain at the procedure site, and it improved within few days	Not reported
**Cervicogenic headache**									
van Zundert 2003 [22] BA / clinical audit	Diagnostic block prior to involvement. Participants with > 50% pain relief received PRF.	*n* = 14	2 months and 6 months after the last patient were included. Mean follow-up was 19.4 months (±8.9 months), maximum 2.5 years.	1.Satisfactory pain relief (GPE: defined as a score of 6 or 7 points on the Likert scale; at least 50% pain relief 2. Duration of the effect 3. Other treatments 4. Change in use of pain medication	Data about pain relief (GPE): 9/14 patients (64%) reported successful pain reduction (6 or 7 points on the GPE Likert scale).	Positive conclusive	No SAEs occurred	No other complications observed	Positive conclusive
Zhang 2011 [23] CR/CR	Diagnostic blocks with 1.5% lidocaine. Positive response was considered as 90% pain relief lasting for 30 min.	*n* = 2	6 months	1. Pain intensity by NRS	**Patient 1:** Baseline NRS: 5; NRS at 6 months: 0. **Patient 2:** Baseline NRS: 4; NRS at 6 months: 0.	Positive inconclusive	No significant complications occurred	No significant complications occurred	Not reported
**Complex regional pain syndrome**									
Albayrak 2016 [24] CR / CS	No other intervention	*n* = 2	1 and 3 days after PRF, 2 and 5 or 10 months (different last follow-up time point for 2 patients)	1. Pain intensity by VAS 2. ROM degree	**Patient 1:** Baseline VAS during movement: 80; VAS at 3 days: 30; VAS at 2 and 10 months: 20. **Patient 2:** Baseline VAS during movement: 100; VAS at 3 days: 30; VAS at 2 months: 20; VAS at 5 months: 10.	Positive inconclusive	No SAEs occurred	No complications were observed	Not reported
Apiliogullari 2015 [25] CR / CR	No other intervention	*n* = 1	1 day after treatment (2 weeks after first PRF the treatment was repeated), 6 months	1. Pain intensity by VAS	Baseline VAS: 100; VAS at 1 day (PRF on L5): 50; VAS at 2 weeks (after repeated PRF on L4): 10. The patient had symptoms relief for over 6 months.	Positive inconclusive	No SAEs occurred	No significant complications occurred	Not reported
**Intractable vertebral metastatic pain**									
Arai 2015 [26] CS / CR	No other intervention	n = 15	0, 1, 7, 21, 28, 35 and 42 days	1. Pain intensity by NRS at rest and while moving	**NRS at rest:** baseline NRS: from 1 to 4 (median 3); NRS at day 1: median 2; NRS at day 7: median 1; NRS at day 21: median 1. Significant decrease in 3 weeks (P < 0.0001). **NRS while moving:** baseline NRS from 5 to 10 (median 8); NRS at day 1: median 4; NRS at day 7: median 4; NRS at day 21: median 3. Significant decrease in 3 weeks (*P* < 0.0001).	Positive conclusive	No SAEs occurred	No other complications occurred	Not reported
**Chronic scrotal and inguinal pain**									
Hofmeester 2013 [27] CR / CR	Diagnostic block with 1 ml of levobupivacaine 0.25%	n = 1	12 months	1. Pain intensity by VAS	Baseline VAS scores: 7–8; VAS initially after intervention: 4; VAS at 12 months: 0–1.	Positive conclusive	Not reported	Not reported	Not reported
**Occipital radiating pain in rheumatoid arthritis**									
Lee 2015 [28] CR / CR	Diagnostic block with 0.3 ml of 0.75% levobupivacaine and 1 mg triamcinolone	n = 1	6 months	1. Pain intensity by VAS	Baseline VAS: 10; VAS at 6 months: 0.	Positive conclusive	Not reported	Not reported	Not reported
**Chronic migraine**									
Li 2018 [29] CR / CR	Diagnostic block with 1 ml of 2% lidocaine	n = 1	1 year	1. Pain intensity by VAS	Baseline VAS: 8; VAS at 1 year: complete pain relief.	Positive inconclusive	Not reported	Not reported	Not reported

*Abbreviations: BA* before and after, *CR* case report, *CS* case series, *CLBP* chronic low back pain, *DRG* dorsal root ganglion, *GPE* global perceived effect, *ICN* intercostal nerves, *MM* medical management, *NRS* numerical rating scale, *ODI* Oswestry disability index, *PaMNI system* patient-mount navigated intervention, *PRF* pulsed radiofrequency, *PRS* pain relief scale *QOLS* quality of life scale, *RCS* retrospective cohort study, *ROM* range of motion, *TENS* transcutaneous electrical nerve stimulation, *SAEs* serious adverse events, *VAS* visual analogue scale, *WOMAC* functional status by Western Ontario and McMaster universities osteoarthritis index

Among 17 included studies there were two randomized controlled trials [14, 15] and 15 non-randomized studies (Table 1). The total number of participants in these studies was 599; the median number of participants was 28 (range: 1 to 127) (Table 1). Both RCTs included participants with low back pain (LBP) [14, 15]. Non-randomized studies included patients with the following indications: LBP [16–18], postsurgical pain [19–21], pain associated with herpes zoster [6], cervicogenic headache [22, 23], complex regional pain syndrome type 1 [24, 25], intractable vertebral metastatic pain [26], chronic scrotal and inguinal pain [27], occipital radiating pain in rheumatoid arthritis [28] and chronic migraine [29] (Table 1). These studies had highly heterogeneous parameters of stimulation (Table 2). Detailed information about inclusion and exclusion criteria, as well as baseline characteristics of included participants, are listed in Table 3.

**Table 2:** Parameters of pulsed radiofrequency treatment of dorsal root ganglion

Author and year	Comparator	Protocol used for treatment	Device used	Position of the electrode
**Low back pain**				
Holanda 2016 [14]	PRF treatment and lidocaine injection vs. laser irradiation	Pulse width: 20 ms with wash-out period of 480 ms; Frequency: 50 Hz; Amplitude: 45 V; Duration: 5 min (with wash out periods of 300 ms); Temperature: 42 °C	150 mm RF probe with 5 mm active tip (company is not specified); COSMAN G4 pulse generator (Cosman Medical, Burlington, MA, USA).	L2
Lee 2018 [15]	Diagnostic block + PRF	Amplitude: 100 V; Duration: 240 s; Temperature: 40–42 °C	20-gauge cannula and Cosman Four-Electrode Radiofrequency Generator (G4) (Cosman Medical, Burlington, MA, USA).	L2 - L5 and S1
Yang 2010 [16]	2 implantation techniques	No stimulation parameters given	22-gauge, SMK-C10 (Radionics Inc., Burlington, MA, USA). RF generator not specified.	L4
Hsu 2017 [17]	No comparator	Frequency: 2 Hz; Amplitude: 45 V; Duration: 120 s; Temperature: 42 °C	10-cm 22-gauge sliced-tip cannula with 1 cm active tip (company is not specified); RF generator (Baylis Medical Company, Montreal, Canada).	L2
Tsou 2010 [18]	No comparator	Frequency: 2 Hz; Amplitude: 45 V; Duration: 120 s; Temperature: 42 °C	10-cm, 22-gauge, curved-tip cannula with a 1 cm active tip electrode (company not specified); RF generator (Baylis Medical Co., Montreal, Canada).	L2-L5 and S1
**Postsurgical pain**				
Albayrak 2017 [19]	TENS + exercise vs. TENS + exercise + PRF	Pulse width: 20 ms active and 480 ms silent periods; Frequency: 2 Hz; Amplitude: 45 V; Duration: 120 s; Temperature: 42 °C	22-gauge cannula (OWL RF cannula 100 mm) with 5 mm active tip electrode (Diros Technology Inc., Canada); NeuroTherm 1100 RF generator (NeuroTherm, Wilmington, MA, USA).	L4
Cohen 2006 [20]	Intercostal nerve stimulation and medical management	Pulse width: 20 ms in 1 s cycle; Frequency: 2 Hz; Amplitude: 45 V; Duration: 120 s; Temperature: 42 °C. The procedure was repeated 4 times, for a total duration of 8 min.	10 cm electrode with a 5 mm active tip (PMC22–100-5, Baylis Medical, Montreal, Quebec, Canada); PMG-115-TD, V2.0A RF generator (Baylis Medical Company, Montreal, Canada).	Exact DRGs are not specified
Fam 2018 [21]	No comparator	Duration: 120 s; Temperature: 42 °C 2 cycles performed	22-gauge, 10 cm, curved cannula with 10 mm active tip (Baylis Medical Company, Montreal, Canada). RF generator used not reported.	T2 and T3
**Pain associated with herpes zoster**				
Kim 2017 [6]	Continuous epidural block (ropivacaine)	Pulse width: 20 ms; Frequency: 2 Hz; Amplitude: 45 V; Duration: 360 s; Temperature: 42 °C	22-gauge 10 cm electrode with 10 mm active tip (Radionics Inc., Burlington, MA, USA); RF generator not specified.	Cervical, thoracic, lumbosacral (exact DRGs not specified)
**Cervicogenic headache**				
van Zundert 2003 [22]	No comparator	Pulse width: 20 ms; Amplitude: 45 V; Duration: 120 s (20 ms current and 480 ms without current); Temperature: 42 °C	54 mm, 22-gauge SMK Pole needle with 4 mm active tip (Cotop International BV, Amsterdam, Netherlands); RFG 3C Plus RF generator (Radionics Inc. Burlington, MA, USA).	Cervical DRG
Zhang 2011 [23]	No comparator	Duration: 360 s; Temperature: 42 °C	Information not given.	C2
**Complex regional pain syndrome**				
Albayrak 2016 [24]	No comparator	Pulse width: 20 ms active and 480 ms silent periods; Frequency: 2 Hz; Amplitude: 40 V; Duration: 120 s; Temperature: 42 °C	22-gauge cannula (OWL RF cannula 54 mm) with 4 mm active tip electrode (Diros Technology Inc., Canada); NeuroTherm 1100 RF generator (NeuroTherm, Wilmington, MA, USA).	C5 and C6
Apiliogullari 2015 [25]	No comparator	Pulse width: 20 ms active and 480 ms silent periods; Frequency: 2 Hz; Amplitude: 45 V; Duration: 120 s; Temperature: 42 °C	22-gauge cannula (OWL RF cannula 54 mm) with 4 mm active tip electrode (Diros Technology Inc., Canada); NeuroTherm 1100 RF generator (NeuroTherm, Wilmington, MA, USA).	L4 and L5
**Intractable vertebral metastatic pain**				
Arai 2015 [26]	No comparator	Pulse width: 20 ms active and 480 ms silent periods; Frequency: 2 Hz; Amplitude: 40 V; Duration: 120 s; Temperature: 42 °C	5 mm active tip KT, guiding needle (Hakko Co. Ltd., Tokyo, Japan); RF generator JK-3 NeuroTherm (Morgan Automation Ltd., Liss, UK).	On each metastatic vertebral body, L1–5 and Th 7, 9–12
**Scrotal and inguinal pain**				
Hofmeester 2013 [27]	No comparator	Pulse width: 8 ms; Frequency: 2 Hz; Amplitude: 45 V; Duration: 480 s; Temperature: 42 °C	Information not given.	T12, L1 and L2
**Occipital radiating pain in rheumatoid arthritis**				
Lee 2015 [28]	No comparator	Duration: 120 s, three cycles performed; Temperature: 42 °C	21-gauge 10 cm insulated needle (company is not specified); RF generator not specified.	C2
**Chronic migraine**				
Li 2018 [29]	No comparator	Pulse width: 20 ms; Frequency: 2 Hz; Amplitude: 45 V; Duration: 900 s; Temperature: 42 °C	22-gauge needle, RF generator G4 (Cosman Medical, Burlington, MA, USA).	C2

*Abbreviations: DRG* dorsal root ganglion; *PRF* pulsed radiofrequency; *RF* radiofrequency

**Table 3:** Inclusion and exclusion criteria and baseline characteristics of participants

ID	Inclusion criteria / Previous treatment	Exclusion criteria	Baseline characteristics
**Low back pain**			
Holanda 2016 [14]	- low back pain for > 3 months	- cancer in lumbar region - coagulation disturbance - infection - neurologic deficits	**PRF treatment group:** - 2 males, 9 females - age range: 42–86 years - pain duration range: 3–144 months **Lidocaine injection group:** - 3 males, 4 females - age range: 33–82 years - pain duration range: 3–48 months **Laser treatment group:** - 3 males, 7 females - age range: 35–84 years - pain duration range: 14–120 months
Lee 2018 [15]	- age 20 years or older - predominantly axial low back pain for > 3 months - medication therapy for > 3 months without benefit - physical rehabilitation for > 3 months without benefit	- an identified etiology of low back pain (i.e., grade II or III spondylolisthesis) - positive response to previous spine interventions such as epidural steroids or sacroiliac joint blocks - previous facet interventions, lumbar spine fusion - untreated coagulopathy - concomitant medical (e.g., unstable angina or degenerative osteoarthritis of knee), or psychiatric conditions - concurrent lumbar pain generator (i.e., muscular/fascial pain, or organs within the abdominal cavity) that could confound the diagnosis of low back pain	**TFESI DRG block + PRF treatment group:** - median age: 74 years, range 53–90 years - median duration of symptoms: 26 months, range: 3–58 months **PRF treatment alone group:** - median age: 75 years, range 33–93 years - median duration of symptoms: 25 months, range: 3–125 months
Yang 2010 [16]	- chronic LBP with focal neurologic symptoms for > 3 months	- spinal disorders - coagulopathy - concomitant medical or psychiatric illness	**PaMNI group:** - 5 males, 11 females - mean age: 55.5 ± 13.9 years **Fluoroscopy group:** - 2 males, 11 females - mean age: 57.2 ± 14.7 years
Hsu 2017 [17]	- age 20 years or older - LBP for > 6 months that worsened upon prolonged sitting or standing - failed to improve after at least 3 months of conservative treatment	- sagittal imbalance - spinal listhesis - infection - tumor - stenosis - disc herniation causing nerve root compression	- 29 males, 55 females - mean age: 56.03 ± 9.04 years
Tsou 2010 [18]	- chronic LBP with or without lower-limb pain for > 6 months - conservative treatment for > 3 months without benefit - participants with symptoms of nerve root compromise due to mild or moderate bulging disc also included	Not given	**LBP without lower limb pain group:** - 26 males, 23 females - men age: 62.94 ± 12.39 years - level treated: L2: 49 **LBP with lower limb pain group:** - 33 males, 45 females - men age: 63.88 ± 14.00 years - levels treated: L2: 78, L3: 14, L4: 33, L5: 72, S1: 21
**Postsurgical pain**			
Albayrak 2017 [19]	- VAS score of ≥3 during activity - pain lasting for ≥2 months - no improvement with physical medicine and rehabilitation - refractory to pharmacological therapies including paracetamol 2 g/day and the maximum tolerable dose of nonsteroidal anti-inflammatory drugs for 1 week and pregabalin 300 mg/day for 2 weeks	- any pathological features, such as acute strain or sprain - stroke/central nervous system disease - serious psychiatric disorders - sciatic pain - fibromyalgia - mental impairment affecting ability to understand tests/measures	**PRF + TENS + exercise group:** - 2 (9.1%) males, 20 (90.9%) females - mean age: 62.1 ± 4.9 years **TENS + exercise group:** - 2 males (11.8%), 15 (88.2%) females - mean age: 65.8 ± 6.5 years
Cohen 2006 [20]	- age 18 years or older - duration of pain ≥3 months - VAS score ≥ 5 - pain deemed to be of neuropathic origin based on history and physical examination	- presence of pathology that could account for a majority of persistent symptoms (e.g. recurrent cancer) - untreated coagulopathy - unstable medical or psychiatric condition	**PRF treatment group:** - 6 males, 7 females - mean age: 45.8 ± 4.7 years **Intercostal nerve stimulation:** - 7 males, 8 females - mean age: 50.8 ± 4.0 years **Medical management group:** - 9 males, 12 females - mean age: 48.6 ± 2.4 years
Fam 2018 [21]	- between 18 and 65 years - refractory to morphine sulfate (MST) and pregabalin	- bleeding tendency - local infection at the site of the intervention - psychological disorders - disturbed anatomy (congenital, traumatic, and postsurgical) - allergy to used medication (local anesthetics and contrast) - inability to lie comfortably during the intervention as the cardiopulmonary distress	Not given
**Pain associated with herpes zoster**			
Kim 2017 [6]	- participants who underwent the procedure between 30 and 180 days after zoster onset	- trigeminal-nerve-involved zoster - follow-up loss within 6 months after the procedure - participants who did not receive appropriate antiviral treatment during the acute phase of herpes zoster - cases where both procedures were performed between 30 and 180 days of zoster onset	**PRF treatment group:** - 11 males, 9 females - mean age: 68.10 ± 7.99 years - days from zoster onset: 68.20 ± 40.53 **Continuous epidural block group:** - 6 males, 16 females - mean age: 70.41 ± 10.25 years - days from zoster onset: 74.09 ± 44.50
**Cervicogenic headache**			
van Zundert 2003 [22]	- 18 years or older - chronic pain in the cervical region for > 6 months - pharmacotherapy, physical or manual therapy, TENS, and/or rehabilitation program without benefit - temporary pain relief of at least 50% on 7-point Likert scale after a diagnostic segmental nerve block - ability to understand the information provided - informed consent	- systemic disease - tumor - clinically demonstrable neurologic deficit - signs of radicular compression	- 5 males, 13 females - age range: 27–77 years - duration of pain prior to treatment: < 1–40 years - DRG level treated: C2: 4, C3: 2, C4: 2, C5: 4, C6: 3, C7: 3
Zhang 2011 [23]	- initial diagnostic selective the greater occipital nerve blocks with 1.5% lidocaine - pain relief of 90% or more lasting for at least 30 min.	NA	**Patient 1:** - 40-years-old woman - pain lasting for 5 years **Patient 2:** - 66-years-old women - pain lasting for 1 year
**Complex regional pain syndrome**			
Albayrak 2016 [24]	- no improvement with the combined use of medical therapy, physical therapy, and the rehabilitation program	NA	**Patient 1**: - 69-years-old women - 9 months of previous pain **Patient 2:** - 48-years-old women
Apiliogullari 2015 [25]	NA	NA	16-years-old girl
**Intractable vertebral metastatic pain**			
Arai 2015 [26]	- confirmed to have vertebral metastases by bone scintigraphy, computed tomography, and magnetic resonance imaging - systemic analgesics did not provide a sound pain relief	- neurological deficit - coagulopathy - significant cardiovascular disease	- 9 males, 6 females - age range: 34–82 years
**Scrotal and inguinal pain**			
Hofmeester 2013 [27]	- an orchidopexy performed - test block of the relevant DRG with 1 ml of levobupivacaine 0.25%	NA	- 13-years-old boy
**Occipital radiating pain in rheumatoid arthritis**			
Lee 2015 [28]	- right 3rd occipital and right 4th, - 5th, and 6th cervical medial branch blocks with levobupivacaine (0.3 mL; 0.75%) and triamcinolone (1 mg) were injected at each level	NA	- 74-years-old female - pain lasting for 2–3 years
**Chronic migraine**			
Li 2018 [29]	- failure of pharmacological therapy and stellate ganglion block - diagnostic C2 block with 1 mL of 2% lidocaine with 75–100% pain relief for only 4 days	NA	- 34-years-old female - 10 years of chronic migraine

*Abbreviations: DRG* dorsal root ganglion, *LBP* low back pain, *NA* not applicable, *PaMNI system* patient-mount navigated intervention, *PRF* pulsed radiofrequency, *RF* radiofrequency, *TENS* transcutaneous electrical nerve stimulation, *TFESI* transforaminal epidural steroid injection, *VAS* visual analogue scale

### Low back pain

In this group, there were 5 studies with a total of 328 participants, including two RCTs with 28 participants in one [14] and 60 participants in another one [15], one retrospective cohort study including 29 participants [16], and two before and after comparisons with 84 participants in one [17] and 127 participants in another [18].

Trial by Holanda et al. [14] included 28 participants which were randomized in three groups: PRF treatment group with the probe directed through the needle in the second lumbar intervertebral foramen (*N* = 11), lidocaine injection group (*N* = 7) and laser irradiation treatment group (*N* = 10). All participants from the lidocaine injection group and laser irradiation group reported a 100% reduction in visual analogue scale (VAS) scores immediately after the treatment, while participants from the PRF group reported a 62.5% reduction in pain. At 1-month follow-up laser irradiation group had a 55.5% reduction in pain; lidocaine injection group 62.5% reduction and PRF group only 20% [14].

An RCT by Lee et al. [15] analyzed predictive value and cost-effectiveness of the use of diagnostic blocks before PRF treatment. They included 60 participants suffering from LBP with or without lower-limb pain, randomized into two groups. In one group (*N* = 30) participants received DRG blocks with 1 ml of 2% bupivacaine and 1 ml of 2% triamcinolone, and those who had at least 50% improvement were scheduled for PRF treatment. The other group (*N* = 30) received only PRF treatment without DRG blocks. Limited low back pain was treated with DRG block or PRF applied to the L2 DRG; lower -limb pain was treated with PRF applied to the L3–S1 DRG. The authors concluded that DRG blocks had no statistically significant impact on the results of PRF treatment, while their application resulted in overall higher medical costs [15].

Yang et al. [16] reported results of a retrospective cohort study that aimed to develop a patient-mounted navigated intervention (PaMNI) system for spinal diseases to evaluate the success of the PRF treatment. The study also included a pilot clinical trial were the new PaMNI system (*N* = 16) was compared to conventional fluoroscopy (*N* = 13). In all patients, PRF treatment was delivered on the L4 DRG. Both groups showed a reduction in VAS scores 1 month after the treatment with no statistically significant difference between groups (*P* = 0.238). However, the study showed the feasibility and efficacy of the PaMNI system [16].

Before and after comparison by Hsu et al. [17] followed 84 participants up to 3 years to investigate the correlation between different types of lumbar lordosis with the outcomes of PRF treatment applied to L2 DRG in chronic low back pain. The analysis showed that after 3-year follow-up participants had a statistically significant reduction in low back pain, regardless of the type of lumbar lordosis [17]. The study by Tsou et al. [18], also followed participants for up to 3 years. They included participants who had low back pain with lower -limb pain (*N* = 78) or without it (*N* = 49). LBP was treated with PRF applied to the L2 DRG and lower-limb pain was treated with PRF applied to the L3–S1 DRG. Percentage of participants achieving at least 50% improvement in VAS scores was similar in both groups at 1-year follow-up, with 20 out of 45 participants (44.44%) in the group without lower -limb pain and 34 out of 74 participants (45.95%) in the group with lower -limb pain [18].

None of the studies reported serious adverse events. Two studies reported minor complications: mild discomfort during the procedure [14] and leakage of the cerebrospinal fluid [17]. One study reported that there were no complications [18]. Two studies from this group did not report any outcomes regarding safety [15, 16], but one of them provided a general warning about the radiation dose exposure [16].

In this group all studies reported positive statements regarding the efficacy of the treatment, four studies had positive conclusive statements [14, 16–18] while one study had positive inconclusive statement [15]. Only one study reported a positive conclusive statement about safety [18], one reported only specific adverse events that occurred [17], while others did not report any conclusion statements (Table 1 and Supplementary Table 3).

### Post-surgical pain

Three studies explored PRF in postsurgical pain, with a total of 188 participants. In a cohort study of Albayrak et al. [19] there were 39 participants with postsurgical pain after total knee arthroplasty. In another cohort study, Cohen et al. [20] included 49 participants suffering from thoracic postsurgical pain. Fam et al. [21] included 100 women suffering from intercostobrachial neuralgia (ICBN) postmastectomy in a study designed as before and after comparison. Despite different etiology of postsurgical pain the majority of participants experienced a reduction in pain after the treatment (details are given in Table 1).

One participant from the study of Cohen et al. [20] had a serious adverse event that could not be related to procedure or treatment. Small pneumothorax was found during a routine scan after the PRF procedure. This participant was treated conventionally and monitored [20]. Pain at the site of the procedure was reported as a mild complication [21]. The third study reported that complications were not observed [19].

Two studies from this group reported positive conclusive statement for efficacy, while the conclusion for safety was not reported [19, 20]. The study by Fam et al. [21] reported positive inconclusive statements for both efficacy and safety [21] (Table 1 and Supplementary Table 3).

### Pain associated with herpes zoster

A retrospective cohort study by Kim et al. [6] with 42 participants addressed PRF of DRG for pain associated with herpes zoster but before post-herpetic neuralgia (PHN) was established. The study analyzed two groups of participants; one received continuous epidural block (*N* = 22), and the other received PRF treatment (*N* = 20) after the acute phase of herpes zoster, but before it was well established, meaning between 30 and 180 days of the herpes zoster diagnosis. Participants from the continuous epidural block group received 0.187% ropivacaine at the rate of 1 ml per hour, while concentration and rate of administration depended on pain relief and adverse effects (mean concentration of ropivacaine and infusion rates used were 0.22 ± 0.07% and 1.82 ± 0.65 ml/hr). When satisfactory pain relief was achieved catheter was removed. Reduction in pain was significantly higher in the PRF group compared to a continuous epidural block group (*P* = 0.029) up to 6 months after the treatment [6]. From the safety aspect, only procedural pain was reported [6]. The study abstract had a positive conclusive statement about efficacy, while safety conclusion was not reported [6] (Table 1 and Supplementary Table 3).

### Cervicogenic headache

The before and after comparison by van Zundert et al. [22] included 18 participants, of which 14 had pain related to non-neuropathic origin (their characteristics were reported separately in Table 1). Participants were followed for a mean time of 19.4 months (maximum follow-up time 2.5 years) [22]. Before study inclusion, participants received diagnostic nerve blocks with 0.5 mL of 2% lidocaine. Treatment outcomes were scored using a 7-point Likert scale.

Participants who had at least 50% pain relief were included in the study and received PRF treatment. Successful PRF treatment was defined as 6 (≥ 50% improvement) or 7 (≥ 75% improvement) points on 7- point Likert scale (Global Perceived Effect good or very good). Participants from the group of non-neuropathic pain origin had successful treatment in 9 cases while treatment was not successful in 5 cases. The case report by Zhang et al. [23] described 2 participants who reported 100% pain relief lasting for 6 months after the treatment. Both studies reported that no complications occurred (Table 1).

The study by van Zundert et al. [22] reported positive conclusive statements about both, safety and efficacy, while Zhang et al. [23] reported positive inconclusive statement about efficacy, while safety was not reported (Table 1 and Supplementary Table 3).

### Complex regional pain syndrome

This group included only two case reports [24, 25] with three participants included. Albayrak et al. [24] reported cases of two women with post-stroke complex regional pain syndrome (CRPS). Both patients used multiple treatment modalities before the PRF treatment, including medical therapy, physical therapy, rehabilitation program and transcutaneous electrical nerve stimulation (TENS). After PRF treatment, both participants had an immediate resolution of their symptoms that lasted up to 5 and 10 months which were final follow-up time points [24].

Apiliogullari et al. [25] reported a case of a 16-year-old girl suffering from CRPS due to sequelae of poliomyelitis, who did not respond to non-steroidal anti-inflammatory drugs. However, after two PRF treatments (first applied at L5 and repeated after 2 weeks at L4 DRG) she reported immediate pain relief, with VAS scores going from 100 points down to 10, this effect remained for over 6 months of follow-up [25]. Both studies reported that no complications occurred (Table 1).

Both studies from this group reported positive inconclusive statements about efficacy, while the conclusion about safety was not reported [24, 25] (Table 1 and Supplementary Table 3).

### Intractable vertebral metastatic pain

The case series of Arai et al. [26] included 15 cases with vertebral metastatic pain, which demonstrated pain relief, defined as a 50% pain reduction from baseline values. Values on the numerical rating scale (NRS), measured during rest and upon movement, were significantly lower 3 weeks after the PRF treatment (*P* < 0.0001) [26]. From the safety aspect, there were no SAEs or other complications (Table 1). The study reported positive conclusive statements about efficacy, while conclusion about safety was not reported [26] (Table 1 and Supplementary Table 3).

### Chronic scrotal and inguinal pain

Hofmeester et al. [27] reported the first case of using PRF to treat scrotal and inguinal pain after orchidopexy in a 13-year boy. PRF was performed at three levels (T12 -L2) after other treatment modalities have failed. The PRF of DRG led to an immediate and lasting pain alleviation of more than 70% as reported by the patient [27]. Information about safety was not reported. The study reported positive conclusive statements about efficacy, while the conclusion about safety was not reported [27] (Table 1 and Supplementary Table 3).

### Occipital radiating pain in rheumatoid arthritis

Lee et al. [28] reported PRF of the C2 DRG to treat occipital radiating headache in a 74-year old woman with rheumatoid arthritis. The patient has not complained of any occipital radiculopathy for 6 months, and the posterior neck pain has since been reduced to a visual analogue scale (VAS) score of three, from initial 6/10. Information about safety were not reported [28]. This study also reported positive conclusive statements about efficacy, while the conclusion about safety was not reported [28] (Table 1 and Supplementary Table 3).

### Chronic migraine

Li et al. [29] reported a case of a 34-year old woman who suffered from chronic migraine with occipital pain. She underwent PRF treatment after the failure of other treatment modalities. The patient had complete pain relief with no symptoms 1 year after the treatment [29]. Details are given in Table 1. The study did not report conclusion about safety, while the conclusion about efficacy was positive inconclusive [29] (Table 1 and Supplementary Table 3).

### Parameters of PRF treatment

Low back pain was a painful condition which had the most different treatment parameters among included studies, with a range of different values for amplitude (45 and 100 V), frequency (2 and 50 Hz) and duration of treatment (120, 240 and 300 s). Pulse width was only reported in one study [14] (Table 2). In other studies parameters were similar, the majority had a pulse width of 20 ms, the amplitude of 45 V, frequency of 2 Hz and duration of 120 s (Table 2). The temperature at the electrode tip was constant parameter, same in all studies, and set to 42 °C in order to avoid tissue damage.

### Participants’ inclusion criteria

More than a half of included studies were before and after comparisons, case series or case reports where participants were included and scheduled for PRF treatment after failure of other treatment modalities and as a last treatment option (Table 3). On the other side, higher-quality studies, such as RCTs and cohort type studies had clearly defined inclusion and exclusion criteria as well as described participants’ baseline characteristics (Table 3).

### Summary on the conclusiveness of the evidence

Among 17 included studies, 11 studies had positive conclusive statements about efficacy; the remaining had positive inconclusive statements. The majority of the studies did not provide conclusive statements regarding safety in the manuscript abstracts. Only three studies provided safety conclusiveness statements – two indicated that the evidence was positive conclusive, and one positive inconclusive (Table 1 and Supplementary Table 3).

### Risk of bias

**Fig. 2:**
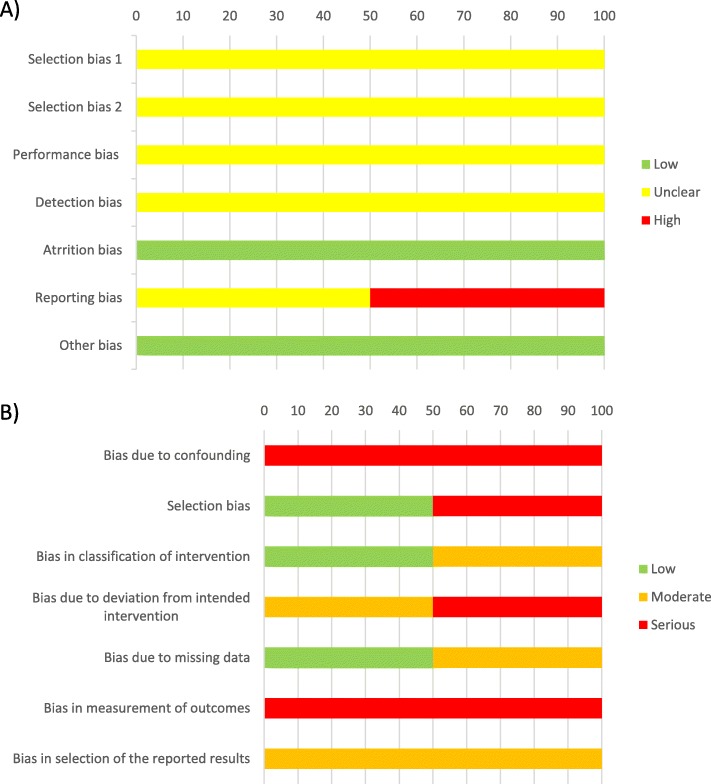
Risk of bias assessment for randomized controlled studies and cohort type studies

Analysis of two included RCTs, with Cochrane Rob tool, indicated that the majority of the domains were judged with unclear RoB due to insufficient information about the used methodology (Supplementary Table 4, Fig. 2). Four non-randomized studies were eligible for assessment with ROBINS-I. The most common judgment for analyzed domains (12 domains out of 28 domains judged for these four studies) was serious RoB. Ten domains were judged with moderate RoB, and only 6 domains with low RoB (Supplementary Table 5, Fig. 2).

### Studies awaiting classification

One RCT, which aims to study DRG thermal RF versus PRF for metastatic pain in the thoracic vertebral body on 69 participants, is classified as completed on July 30, 2018. Results were reported to Clinical Trials.gov but were returned to the authors after the quality control review so results are still not publicly available (NCT03204942). A trial that aimed to study superior hypogastric plexus block versus PRF for chronic pelvic cancer pain on 40 participants is classified as ‘Not yet recruiting’ since June 26, 2018 (NCT03228316). Studies awaiting classification are described in Supplementary Table 6.

## Discussion

In this systematic review, we included 17 studies about the treatment of several non-neuropathic chronic pain conditions with PRF directed to DRG. All studies presented positive conclusions (both conclusive and inconclusive) about the efficacy of the treatment. However, the studies were mostly non-randomized, with small sample sizes, and issues related to the risk of bias. Therefore, their results should only be considered as preliminary.

PRF was developed as a less destructive pain relief modality alternative to conventional radiofrequency (CRF) which can selectively block delta and C fibers [30]. The first report about the clinical effects of PRF on DRG was published relatively recently, in 1998. Due to its theoretical benefits, it was postulated that PRF could be particularly helpful in neuropathic pain [31]. However, we have observed in the literature that clinicians and researchers apply PRF to non-neuropathic chronic pain as well.

Despite the number of studies found in the literature about the treatment of non-neuropathic chronic pain in humans with PRF, their findings cannot be generalized. In the studies that we have found, the PRF was usually initiated after other treatments have failed. We reported a similar issue in our recent systematic review in which we studied the efficacy and safety of EFS of DRG [12]. In that systematic review, we found only one RCT among 29 included studies; most of the studies were low-level of evidence – non-randomized study designs, including case series and case reports. The review about EFS of DRG also included few participants, with a median of 6 participants per study [12]. In this systematic review there were 17 included studies, with a median of 28 participants.

The paucity of large and high-quality studies in the field of DRG neuromodulation is likely due to the relative novelty of this approach for the treatment of pain. In this systematic review, about PRF of DRG in non-neuropathic pain, only two of 17 included studies were RCTs, and RoB judgment for the majority of their methodological aspects was unclear. Likewise, the most common assessment in non-randomized studies assessed with the ROBINS-I tool was that there was a serious risk of bias. Besides their suboptimal methodological reporting, the analyzed studies were relatively small. Even the two included randomized controlled trials were small; one included a total of 28 patients in 3 groups, and the other one included 60 patients in two groups.

The highest number of studies was found for the low back pain indication. However, we were not able to perform a meta-analysis due to clinical heterogeneity of the studies, as can be seen from characteristics of included studies, different comparators used in included trials, and different stimulation parameters. Differences in treatment approaches can result in different clinical outcomes.

Despite the low level of evidence, all of the analyzed studies sent positive conclusions to the research community in their abstracts. The majority of these conclusions were conclusive, i.e. they did not mention the need to conduct further studies on this subject. Despite the authors’ positive conclusions regarding the tested interventions, caution is needed when advising DRG targeted PRF to chronic pain patients, because of the paucity of high-quality and high-level evidence. This intervention should be tested in large-scale, high-quality RCTs to truly test whether the intervention has expected benefits and harms. Until then, these studies should be treated as preliminary evidence only.

A broad focus of this systematic review could be considered as a limitation of this review, as we included any pain condition that fits the IASP criteria of non-neuropathic pain. Furthermore, we acknowledge that the examined studies included patients with various clinical conditions, and thus there is a possibility that the effectiveness of the treatment depends on underlying pathogenic mechanisms. However, as can be observed from our results, there were very few studies in each group of indications; the highest number of studies (five) was found for low back pain. Therefore, focusing on every single one of these indications in a separate systematic review would result in a high number of systematic reviews, with minimal results included. Furthermore, with this approach, we are giving readers a very wide and informative picture of all the non-neuropathic pain conditions that were reported in the literature as treated with DRG targeted PRF.

We have used IASP classification for definitions of non-neuropathic pain; these classifications are evolving and changing, so the included conditions may be categorized differently, depending on the time of categorization and reference classification used. Previous versions of chronic pain classification were to some extent insufficient for chronic neuropathic pain conditions since some conditions were not defined properly or were missing so we decided to use the updated version of classification since it is crucial to get the comprehensive evidence synthesis. According to the newest IASP classification that we used (ICD-11) when deciding about study inclusion we might have included some studies that in previous versions of classification were classified either as neuropathic pain or as the pain of mixed origin. We have included CRPS 1 [25], which is not considered neuropathic pain. In the study of Kim et al. [6] the authors studied the effects of DRG PRF beyond the acute phase of zoster, bur before PHN was well established (from 30 days to 180 days after zoster onset). The study of van Zundert [22] has excluded “signs of radicular compression”.

It has been questioned before what is the value of systematic review including poor evidence and small studies [32]. However, such systematic reviews are valuable because they are highlighting the paucity of evidence and the low quality of available information [33]. Our systematic review is such a case. We even included two case reports with only one participant which may be considered anecdotal rather than firm evidence. It could be argued that such studies should not even be included in systematic reviews; however, we did not set any restrictions regarding number of participants in our study eligibility criteria. By showing that many clinicians and researchers have published small studies, with low-level evidence, about potential benefits of PRF in chronic non-neuropathic pain, we hope that trialists will be inspired to explore this intervention in studies that are considered high-level evidence.

## Conclusion

Even though PRF of DRG was primarily studied for neuropathic pain, we have found as many as 17 published studies that have reported the use of DRG targeted PRF in non-neuropathic pain conditions. Although all of these studies reported positive information regarding the analyzed interventions, considerable caution is needed when interpreting these results as anything more than preliminary. The quality of evidence is low, as there were only two randomized controlled trials among included studies, and the risk of bias was predominantly unclear in RCTs and severe among non-randomized studies. The majority of studies included patients that have failed other therapies so these results cannot be generalized. PRF treatment needs to be tested in new, high-quality and large-scale trials, to confirm the efficacy of this intervention.

## Supplementary information

**Additional file 1: Supplementary Table 1.** Search strategies for four bibliographic databases searched.

**Additional file 2: Supplementary Table 2**. Characteristics of excluded studies.

**Additional file 3: Supplementary Table 3.** Conclusion statements presented in the abstracts of included studies.

**Additional file 4: Supplementary Table 4.** Individual Cochrane risk of bias judgments for randomized controlled trials.

**Additional file 5: Supplementary Table 5.** Individual ROBINS judgments for non-randomized studies.

**Additional file 6: Supplementary Table 6.** Details about studies awaiting classification.

## Data Availability

The datasets used and/or analyzed during the current study are available from the corresponding author on reasonable request.

## Abbreviations

AEs: adverse events
BA: before and after comparison
CLBP: chronic low back pain
CR: case report
CRPS: complex regional pain syndrome
CRD: center for reviews and dissemination
CS: case series
DRG: dorsal root ganglion
EFS: electrical field stimulation
GPE: global perceived effect IASP – International Association for the Study of Pain
ICN: intercostal nerves
LBP: low back pain
MM: medical management
NA: not applicable
NRS: numeric rating scale
NRSD: non-randomized study designs
ODI: Oswestry disability index
PaMNI: patient-mounted navigated intervention
PHN: post-herpetic neuralgia
PRF: pulsed radiofrequency
QOLS: quality of life scale
RCS: retrospective cohort study
RCT: randomized controlled trial
RoB: risk of bias
ROBINS-I: Risk of Bias In Non-randomized Studies of Interventions
ROM: range of motion
SAEs: serious adverse events
TENS: transcutaneous electrical nerve stimulation
TFESI: transforaminal epidural steroid injection
VAS: visual analogue scale
WHO ICTRP: World Health Organization’s International Clinical Trial Registry Platform
WOMAC: functional status by Western Ontario and McMaster universities osteoarthritis index
