# Supplementary material for: Efficacy and safety of pulsed radiofrequency as a method of dorsal root ganglia stimulation for treatment of non-neuropathic pain: a systematic review
Source: BMC Anesthesiol. 2020 May 4;20:105. doi: 10.1186/s12871-020-01023-9 (PMC7199300; doi:10.1186/s12871-020-01023-9)
Supplement: Supplementary file 1 — Additional file 1: Supplementary Table 1. Search strategies for four bibliographic databases searched. [file 12871_2020_1023_MOESM1_ESM.docx]

**2. Embase (via OVID)**

**Intervention**

1) exp electrotherapy/

2) electrostimulation/

3) electrode implant/

4) neuromodulat$.ti,ab,kw.

5) neurostimulat$.ti,ab,kw.

6) stimulat$.ti,ab,kw.

7) electrod$ implant$.ti,ab,kw.

8) implantable neurostimulator/

9) OR/1-8

**Location**

10) exp spinal ganglion/

11) spinal gangli$.ti,ab,kw.

12) sensory gangli$.ti,ab,kw.

13) dorsal root gangli$.ti,ab.

14) DRG$.ti,ab,kw.

15) OR/10-14

16) 9 AND 15

**Condition**

17) exp pain/

18) pain$.ti,ab,kw.

19) analgesia/

20) analgesi$.ti,ab.

21) OR/17-20

22) 16 AND 21

**3. Psych INFO (via EBSCO host)**

1. „electric stimulation therapy“ OR neurostimulat* OR neuromodulat* or stimulat* OR

„electrod* implant* OR „implantable neurostimulator“ (TI)

2. electric stimulation therapy“ OR neurostimulat* OR neuromodulat* or stimulat* OR

„electrod* implant* OR „implantable neurostimulator“ (AB)

3. electric stimulation therapy“ OR neurostimulat* OR neuromodulat* or stimulat* OR

„electrod* implant* OR „implantable neurostimulator“ (KW)

4. „sensory gangli*“ OR „spinal gangli*“ OR „dorsal root gangli* OR DRG (TI)

5. „sensory gangli*“ OR „spinal gangli*“ OR „dorsal root gangli* OR DRG (AB)

6. „sensory gangli*“ OR „spinal gangli*“ OR „dorsal root gangli* OR DRG (KW)

7. pain* OR analgesi* (TI)

8. pain* OR analgesi* (AB)

9. pain* OR analgesi* (KW)

**4. CINAHL (via EBSCO host)**

1. (MM &quot;Electric Stimulation&quot;) OR (MM &quot;Electrodes, Implanted&quot;) OR &quot;electrod* implant*&quot; OR

&quot;neurostimulat*&quot; OR &quot;neuromodulat*&quot; OR &quot;stimulat*&quot; OR &quot;implant* neurostimulat*&quot;

2. (MM &quot;Ganglia, Sensory&quot;) OR &quot;sensory gangli*&quot; OR &quot;spinal gangli*&quot; OR &quot;dorsal root gangli*&quot;

OR &quot;DRG*&quot;

3. (MH &quot;Pain+&quot;) OR &quot;pain*&quot; OR (MM &quot;Analgesia&quot;) OR &quot;analgesi*&quot;

4. 1 AND 2 AND 3
