# Supplementary material for: Efficacy and safety of pulsed radiofrequency as a method of dorsal root ganglia stimulation for treatment of non-neuropathic pain: a systematic review
Source: BMC Anesthesiol. 2020 May 4;20:105. doi: 10.1186/s12871-020-01023-9 (PMC7199300; doi:10.1186/s12871-020-01023-9)
Supplement: Supplementary file 2 — Additional file 2: Supplementary Table 2. Characteristics of excluded studies. [file 12871_2020_1023_MOESM2_ESM.docx]

**Supplementary table 2. Characteristics of excluded studies**

|  | **Study** | **Reason for exclusion** |
| --- | --- | --- |
|  | Abejon 2007 [1] | Patients with neuropathic pain |
|  | Chang 2017a [2] | Patients with neuropathic pain |
|  | Chang 2017b [3] | Patients with neuropathic pain |
|  | Chao 2008 [4] | Patients with neuropathic pain |
|  | Choi 2011 [5] | Patients with neuropathic pain |
|  | Choi 2012 [6] | Patients with neuropathic pain |
|  | Das 2018 [7] | Patients with neuropathic pain |
|  | Egorov 2015 [8] | Patients with neuropathic pain |
|  | Esparza-Minana 2018 [9] | Patients with neuropathic pain |
|  | Gabrhelik 2007 [10] | Patients with neuropathic pain |
|  | Halim 2017 [11] | Nerve roots were exposed to PRF |
|  | Hunter 2017 [12] | PRF was not used as therapeutic method for treatment of chronic pain |
|  | Ke 2013 [13] | PRF electrode positioned to the intercostal nerve |
|  | Kim 2008 [14] | Patients with neuropathic pain |
|  | Kim 2017 [15] | Patients with neuropathic pain |
|  | Kim 2018 [16] | Patients with neuropathic pain |
|  | Koh 2015 [17] | Patients with neuropathic pain |
|  | Lee 2011 [18] | PRF of non-nervous, soft tissue (not DRG) |
|  | Lee 2016 [19] | Patients with neuropathic pain |
|  | Lee 2018 [20] | Patients with neuropathic pain |
|  | Lin 2014 [21] | Patients with neuropathic pain |
|  | Lindquist 2016 [22] | DRG or peripheral nerve were targeted for PRF |
|  | Makharita 2015 [23] | Nerve roots were exposed to PRF |
|  | Martin 2007 [24] | Different anatomic structures targeted for PRF: sacroiliac and shoulder joint, DRG and ventral rami, stellate ganglion treated (no separate pain intensity scores for DRG) |
|  | Mehta 2017 [25] | Patients with neuropathic pain |
|  | Munglani 1999 [26] | Stimulation of spinal roots and/or DRG |
|  | Simopoulos 2008 [27] | Stimulation of segmental nerve too, no pain intensity data about each stimulated segment |
|  | Shanthanna 2014 [28] | Patients with neuropathic pain |
|  | Sluijter 1998 [29] | Patients with neuropathic pain |
|  | Surbano 2016 [30] | Patients with neuropathic pain |
|  | Teixeira 2005 [31] | Stimulation of segmental nerve too, no pain intensity data about each stimulated segment |
|  | Trinidad 2015 [32] | Combined interventions without separate results for PRF treatment |
|  | van Boxem 2011 [33] | Patients with neuropathic pain |
|  | Van Boxem 2015 [34] | Patients with neuropathic pain |
|  | Van Boxem 2016 [35] | Parallel intervention of diagnostic block (lidocaine) and PRF treatment, S1 level was also stimulated, there was no separate pain intensity data |
|  | Van Zundert 2007 [36] | Patients with neuropathic pain |
|  | Van Wijk 2000 [37] | High temperature PRF |
|  | Vigneri 2014 [38] | Patients with neuropathic pain |
|  | Vigneri 2017 [39] | Patients with neuropathic pain |
|  | Wan 2015 [40] | Nerve roots were exposed to PRF |
|  | Wan 2016 [41] | Patients with neuropathic pain |
|  | Wang 2017 [42] | Nerve roots were exposed to PRF |
|  | Xiao 2015 [43] | Combined PRF and selective cervical nerve root block therapy |
|  | Xie 2016 [44] | High temperature PRF |
|  | Yoon 2014 [45] | Patients with neuropathic pain |
|  | Zeldin 2008 [46] | Poster presentation |

**Abbreviations**: DRG = dorsal root ganglion; PRF = pulsed radiofrequency

**References**

1. Abejon D, Garcia-Del-Valle S, Fuentes ML, Gomez-Arnau JI, Reig E, Van Zundert J. Pulsed radiofrequency in lumbar radicular pain: clinical effects in various etiological groups*.* *Pain practice : the official journal of World Institute of Pain* 7(1), 21-26 (2007).

2. Chang MC, Cho YW, Ahn SH. Comparison between bipolar pulsed radiofrequency and monopolar pulsed radiofrequency in chronic lumbosacral radicular pain: A randomized controlled trial*.* *Medicine (United States)* 96 (9) (no pagination)(e6236), (2017).

3. Chang MC, Cho YW. Effects of pulsed radiofrequency on spasticity in patients with spinal cord injury: a report of two cases*.* *Neural Regeneration Research* 12(6), 977-980 (2017).

4. Chao S-C, Lee H-T, Kao T-H *et al*. Percutaneous pulsed radiofrequency in the treatment of cervical and lumbar radicular pain*.* *Surgical Neurology* 70(1), 59-65 (2008).

5. Choi GS, Ahn SH, Cho YW, Lee DK. Short-term effects of pulsed radiofrequency on chronic refractory cervical radicular pain*.* *Annals of rehabilitation medicine* 35(6), 826-832 (2011).

6. Choi G-S, Ahn S-H, Cho Y-W, Lee D-G. Long-Term Effect of Pulsed Radiofrequency on Chronic Cervical Radicular Pain Refractory to Repeated Transforaminal Epidural Steroid Injections*.* *Pain Medicine* 13(3), 368-375 (2012).

7. Das B, Conroy M, Moore D, Lysaght J, Mccrory C. Human dorsal root ganglion pulsed radiofrequency treatment modulates cerebrospinal fluid lymphocytes and neuroinflammatory markers in chronic radicular pain*.* *Brain. Behav. Immun.* 70 157-165 (2018).

8. Egorov OE, Evzikov GY. Repeated radiofrequency dorsal root ganglion neurotomy in a patient with painful radiculopathy. A case report and literature review*.* *Nevrologicheskii zhurnal* 20(1), 28-33 (2015).

9. Esparza-Minana JMM, G.; Belaouchi, M.; Cort-Martinez, L.; Robert-Sanchez, L.; Vicedo Lillo, R. Pulsed radiofrequency of the thoracic dorsal root ganglion with epidural catheter-electrode. A new approach for an effective treatment*.* *Revista de la Sociedad Española del Dolor* 25(1), 45-50 (2018).

10. Gabrhelik T, Michalek P, Berta E, Adamus M, Pieran M, Dolecek L. Pulsed Radiofrequency of radicular pain*.* *Ceska a Slovenska Neurologie a Neurochirurgie* 70(5), 533-537 (2007).

11. Halim W, Van Der Weegen W, Lim T, Wullems JA, Vissers KC. Percutaneous Cervical Nucleoplasty vs. Pulsed Radio Frequency of the Dorsal Root Ganglion in Patients with Contained Cervical Disk Herniation; A Prospective, Randomized Controlled Trial*.* *Pain Practice* 17(6), 729-737 (2017).

12. Hunter CW, Yang A, Davis T. Selective Radiofrequency Stimulation of the Dorsal Root Ganglion (DRG) as a Method for Predicting Targets for Neuromodulation in Patients With Post Amputation Pain: A Case Series*.* *Neuromodulation.*  (2017).

13. Ke M, Yinghui F, Yi J *et al*. Efficacy of pulsed radiofrequency in the treatment of thoracic postherpetic neuralgia from the angulus costae: A randomized, double-blinded, controlled trial*.* *Pain physician* 16(1), 15-25 (2013).

14. Kim YH, Lee CJ, Lee SC *et al*. Effect of pulsed radiofrequency for postherpetic neuralgia*.* *Acta Anaesthesiologica Scandinavica* 52(8), 1140-1143 (2008).

15. Kim K, Jo D, Kim E. Pulsed Radiofrequency to the Dorsal Root Ganglion in Acute Herpes Zoster and Postherpetic Neuralgia*.* *Pain physician* 20(3), E411-E418 (2017).

16. Kim SJ, Park SJ, Yoon DM, Yoon KB, Kim SH. Predictors of the analgesic efficacy of pulsed radiofrequency treatment in patients with chronic lumbosacral radicular pain: a retrospective observational study*.* *Journal of pain research* 11 1223-1230 (2018).

17. Koh W, Choi S-S, Karm MH *et al*. Treatment of chronic lumbosacral radicular pain using adjuvant pulsed radiofrequency: A randomized controlled study*.* *Pain Medicine* 16(3), 432-441 (2015).

18. Lee JS, Yoon KB, Kim IK, Yoon DM. Pulsed radiofrequency treatment of pain relieving point in a soft tissue*.* *Korean Journal of Pain* 24(1), 57-59 (2011).

19. Lee DG, Ahn SH, Lee J. Comparative Effectivenesses of Pulsed Radiofrequency and Transforaminal Steroid Injection for Radicular Pain due to Disc Herniation: A Prospective Randomized Trial*.* *J. Korean Med. Sci.* 31(8), 1324-1330 (2016).

20. Lee DG, Cho YW, Ahn SH, Chang MC. The Effect of Bipolar Pulsed Radiofrequency Treatment on Chronic Lumbosacral Radicular Pain Refractory to Monopolar Pulsed Radiofrequency Treatment*.* *Pain physician* 21(2), E97-E103 (2018).

21. Lin WL, Lin BF, Cherng CH *et al*. Pulsed radiofrequency therapy for relieving neuropathic bone pain in cancer patients*.* *Journal of Medical Sciences (Taiwan)* 34(2), 84-87 (2014).

22. Lindquist J, Backryd E. Pulsed radiofrequency in clinical practice - A retrospective analysis of 238 patients with chronic non-cancer pain treated at an academic tertiary pain centre*.* *Scandinavian Journal of Pain* 12 68-73 (2016).

23. Makharita MY, Amr YM. Pulsed radiofrequency for chronic inguinal neuralgia*.* *Pain physician* 18(2), E147-E155 (2015).

24. Martin DC, Willis ML, Mullinax LA, Clarke NL, Homburger JA, Berger IH. Pulsed radiofrequency application in the treatment of chronic pain*.* *Pain Practice* 7(1), 31-35 (2007).

25. Mehta V, Snidvongs S, Ghai B, Langford R, Wodehouse T. Characterization of peripheral and central sensitization after dorsal root ganglion intervention in patients with unilateral lumbosacral radicular pain: a prospective pilot study*.* *British Journal of Anaesthesia* 118(6), 924-931 (2017).

26. Munglani R. The longer term effect of pulsed radiofrequency for neuropathic pain*.* *Pain* 80(1-2), 437-439 (1999).

27. Simopoulos TT, Kraemer J, Nagda JV, Aner M, Bajwa ZH. Response to Pulsed and Continuous Radiofrequency Lesioning of the Dorsal Root Ganglion and Segmental Nerves in Patients with Chronic Lumbar Radicular Pain*.* *Pain physician* 11(2), 137-144 (2008).

28. Shanthanna H, Chan P, Mcchesney J, Thabane L, Paul J. Pulsed radiofrequency treatment of the lumbar dorsal root ganglion in patients with chronic lumbar radicular pain: A randomized, placebo-controlled pilot study*.* *Journal of pain research* 7 47-55 (2014).

29. Sluijter ME, Cosman ER, Rittmann WB, Van Kleef M. The effects of pulsed radiofrequency fields applied to the dorsal root ganglion - a preliminary report*.* *Pain Clinic* 11(2), 109-117 (1998).

30. Surbano M, Cristiani F, Ayala S, Castromán P. Radiofrecuencia pulsada del ganglio de la raíz dorsal en un caso de sindrome radicular lumbosacro crónico refractario a los esteroides epidurales. chronic lumbosacro syndrome refractory to epidural steroids*.* *Anestesia Analgesia Reanimación* 29(2), 18-30 (2016).

31. Teixeira A, Grandinson M, Sluijter ME. Pulsed radiofrequency for radicular pain due to a herniated intervertebral disc--an initial report*.* *Pain practice : the official journal of World Institute of Pain* 5(2), 111-115 (2005).

32. Trinidad JM, Carnota AI, Failde I, Torres LM. Radiofrequency for the Treatment of Lumbar Radicular Pain: Impact on Surgical Indications*.* *Pain Research and Treatment* doi:10.1155/2015/392856 (2015).

33. Van Boxem K, Van Bilsen J, De Meij N *et al*. Pulsed Radiofrequency Treatment Adjacent to the Lumbar Dorsal Root Ganglion for the Management of Lumbosacral Radicular Syndrome: A Clinical Audit*.* *Pain Medicine* 12(9), 1322-1330 (2011).

34. Van Boxem K, De Meij N, Kessels A, Van Kleef M, Van Zundert J. Pulsed Radiofrequency for Chronic Intractable Lumbosacral Radicular Pain: A Six-Month Cohort Study*.* *Pain Medicine* 16(6), 1155-1162 (2015).

35. Van Boxem K, De Meij N, Patijn J *et al*. Predictive factors for successful outcome of pulsed radiofrequency treatment in patients with intractable lumbosacral radicular pain*.* *Pain Medicine (United States)* 17(7), 1233-1240 (2016).

36. Van Zundert J, Patijn J, Kessels A, Lame I, Van Suijlekom H, Van Kleef M. Pulsed radiofrequency adjacent to the cervical dorsal root ganglion in chronic cervical radicular pain: A double blind sham controlled randomized clinical trial*.* *Pain* 127(1-2), 173-182 (2007).

37. Van Wijk RMaW, Geurts JWM, Buijs EJ. Criteria for electrical nerve stimulation and outcome of radiofrequency treatment of the dorsal root ganglion in the lower back for chronic pain*.* *Pain Clinic* 12(4), 281-286 (2000).

38. Vigneri S, Sindaco G, Gallo G *et al*. Effectiveness of Pulsed Radiofrequency with Multifunctional Epidural Electrode in Chronic Lumbosacral Radicular Pain with Neuropathic Features*.* *Pain Physician* 17(6), 477-486 (2014).

39. Vigneri S, Sindaco G, Zanella M, Sette E, Tugnoli V, Pari G. Interventional treatment for neuropathic pain due to combined cervical radiculopathy and carpal tunnel syndrome: a case report*.* *Clinical Case Reports* 5(4), 414-418 (2017).

40. Wan C-F, Zhang L, Xi Q, Song T. Clinical Efficacy of the Pulsed Radiofrequency on Primary Glossopharyngeal Neuralgia*.* *Journal of China Medical University* 44(9), 780-782 (2015).

41. Wan CF, Liu Y, Dong DS *et al*. Bipolar High-Voltage, Long-Duration Pulsed Radiofrequency Improves Pain Relief in Postherpetic Neuralgia*.* *Pain Physician* 19(5), E721-E728 (2016).

42. Wang F, Zhou Q, Xiao L *et al*. A Randomized Comparative Study of Pulsed Radiofrequency Treatment With or Without Selective Nerve Root Block for Chronic Cervical Radicular Pain*.* *Pain practice : the official journal of World Institute of Pain* 17(5), 589-595 (2017).

43. Xiao LZ, Li J, Li DS *et al*. A posterior approach to cervical nerve root block and pulsed radiofrequency treatment for cervical radicular pain: a retrospective study*.* *J. Clin. Anesth.* 27(6), 486-491 (2015).

44. Xie G-L, Guo D-P, Li Z-G, Liu C, Zhang W. Application of radiofrequency thermocoagulation combined with adriamycin injection in dorsal root ganglia for controlling refractory pain induced by rib metastasis of lung cancer (a STROBE-compliant article)*.* *Medicine (Hagerstown)* 95(40), e4785 (2016).

45. Yoon YM, Han SR, Lee SJ, Choi CY, Sohn MJ, Lee CH. The efficacy of pulsed radiofrequency treatment of cervical radicular pain patients*.* *Korean Journal of Spine* 11(3), 109-112 (2014).

46. Zeldin A, Ioscovich A. Pulsed Radiofrequency for Metastatic Pain Treatment*.* *Pain physician* 11(6), 921-922 (2008).
