# Supplementary material for: Efficacy and safety of pulsed radiofrequency as a method of dorsal root ganglia stimulation for treatment of non-neuropathic pain: a systematic review
Source: BMC Anesthesiol. 2020 May 4;20:105. doi: 10.1186/s12871-020-01023-9 (PMC7199300; doi:10.1186/s12871-020-01023-9)
Supplement: Supplementary file 3 — Additional file 3: Supplementary Table 3. Conclusion statements presented in the abstracts of included studies. [file 12871_2020_1023_MOESM3_ESM.docx]

**Supplementary table 3**. **Conclusion statements presented in the abstracts of included studies**

| **Study ID** | **Study design** | **Conclusion statement for efficacy** | | **Category for efficacy** | **Conclusion statement for safety** | **Category for safety** |
| --- | --- | --- | --- | --- | --- | --- |
| **Low back pain** | | | | | | |
| Holanda 2016 [[1](#_ENREF_1)] | RCT | “Both lidocaine injection and laser irradiation were more effective than radiofrequency treatment for immediate and longer term (1 month post-treatment) chronic back pain.” | | Positive conclusive | Not reported | Not reported |
| Lee 2018 [[2](#_ENREF_2)] | RCT | “In this comparative cost-effectiveness study, the application of diagnostic DRG blocks before DRG-PRF did not have a significant impact on patient satisfaction, pain index score, or pain medication reduction.” | | Positive inconclusive | Not reported | Not reported |
| Yang 2010 [[3](#_ENREF_3)] | Retrospective cohort study | “The clinical trial of PRF- DRG treatment showed the clinical feasibility and efficacy of this system.” | | Positive conclusive | Not reported | Not reported |
| Hsu 2017 [[4](#_ENREF_4)] | Before and after comparison | “PRF applied to the L2 DRG is an alternative procedure for treating patients with chronic low back pain, regardless of which type of LL the patients have.” | | Positive conclusive | “Two patients experienced cerebrospinal fluid leakage when the needle was moved toward the L2 DRG, but neither patient experienced a neurological deficit.” | Specific adverse events mentioned, no overall conclusion about safety |
| Tsou 2010 [[5](#_ENREF_5)] | Before and after comparison | “The results of this prospective analysis showed that treatment with pulsed radiofrequency applied at the L-2 DRG is safe and effective for treating chronic low-back pain.”  ”The results indicate that pulsed radiofrequency provided intermediate-term relief of low-back pain.” | | Positive conclusive | “The results of this prospective analysis showed that treatment with pulsed radiofrequency applied at the L-2 DRG is safe and effective for treating chronic low-back pain.” | Positive conclusive |
| **Postsurgical pain** | | | | | | |
| Albayrak 2017 [[6](#_ENREF_6)] | Prospective cohort study | “Adding PRF to TENS and exercise therapy is useful in reducing the degree of pain and the neuropathic component of PPSP in patients with PPSP” | | Positive conclusive | Not reported | Not reported |
| Cohen 2006 [[7](#_ENREF_7)] | Retrospective cohort study | “Pulsed RF of the DRG was a superior treatment to pharmacotherapy and pulsed RF of the ICN in patients with CPTP.” | | Positive conclusive | Not reported | Not reported |
| Fam 2018 [[8](#_ENREF_8)] | Before and after comparison | “PRF and steroid injection on T2 and T3 DRGs assumed an effective and safe method for ICBN postmastectomy treatment.” | | Positive inconclusive | “PRF and steroid injection on T2 and T3 DRGs assumed an effective and safe method for ICBN postmastectomy treatment.” | Positive inconclusive |
| **Pain associated with herpes zoster** | | | | | | |
| Kim 2017 [[9](#_ENREF_9)] | Retrospective cohort study | “This study revealed that DRG PRF was more effective than a continuous  epidural block in treating ZAP after the acute phase of zoster. | Positive conclusive | | Not reported | Not reported |
| **Cervicogenic headache** | | | | | | |
| van Zundert 2003 [[10](#_ENREF_10)] | Before and after comparison | “Satisfactory pain relief of at least 50% was achieved in 13 of 18 (72%) patients at 8 weeks. More than one year after treatment, six patients (33%) continue to rate treatment outcome as good or very good.” | Positive conclusive | | “None of the patients reported post-treatment neuritis or other adverse events.”  ”No side effects were reported.” | Positive conclusive |
| Zhang 2011 [[11](#_ENREF_11)] | Case report | “This case study demonstrates the effectiveness of PRF to treat cervicogenic headache originating from the C2 nerve. However, we need to further evaluate the results using more samples.” | Positive inconclusive | | Not reported | Not reported |
| **Complex regional pain syndrome** | | | | | | |
| Albayrak 2016 [[12](#_ENREF_12)] | Case report | “Pulsed radiofrequency applied to cervical dorsal root ganglia might play a significant role in multi-modal approach of complex regional pain syndrome type 1 management after stroke” | Positive inconclusive | | Not reported | Not reported |
| Apiliogullari 2015 [[13](#_ENREF_13)] | Case report | “This case illustrates that PRF applied to lumbar 4 and lumbar 5 DRG may play a significant role in CRPS type 1 management after the surgical treatment of poliomyelitis sequelae in adolescent patients” | Positive inconclusive | | Not reported | Not reported |
| **Intractable vertebral metastatic pain** | | | | | | |
| Arai 2015 [[14](#_ENREF_14)] | Case series | “DRG pulsed RF procedure provided sound pain relief for patients with intractable vertebral metastatic pain” | Positive conclusive | | Not reported | Not reported |
| **Chronic scrotal and inguinal pain** | | | | | | |
| Hofmeester 2013 [[15](#_ENREF_15)] | Case report | “After many therapies had failed, we treated the dorsal root ganglia of thoracic 12, lumbar 1 and lumbar 2 with pulsed radiofrequency, finally resulting in alleviation of his pain.” | Positive conclusive | | Not reported | Not reported |
| **Occipital radiating pain in rheumatoid arthritis** | | | | | | |
| Lee 2015 [[16](#_ENREF_16)] | Case report | “Here, we report the successful treatment of a RA patient with occipital radiating headache using pulsed radiofrequency therapy at the C2 dorsal root ganglion.” | Positive conclusive | | Not reported | Not reported |
| **Chronic migraine** | | | | | | |
| Li 2018 [[17](#_ENREF_17)] | Case report | “This suggests that C2 DRG PRF might be considered as an alternative treatment for chronic migraine with occipital pain.” | Positive inconclusive | | Not reported | Not reported |

**Abbreviations:** CRPS = complex regional pain syndrome; CPTP = chronic postsurgical thoracic pain; DRG = dorsal root ganglion; ICBN = intercostobrachial neuralgia; ICN = intercostal nerves; LL = lumbar lordosis; PPSP = persistent postsurgical pain; PRF = pulsed radiofrequency; RA = rheumatoid arthritis; RCT = randomized controlled trial; RF = radiofrequency; TENS = transcutaneous electrical nerve stimulation; ZAP = zoster-related pain.

**References**

1. Holanda VM, Chavantes MC, Silva DF *et al*. Photobiomodulation of the dorsal root ganglion for the treatment of low back pain: A pilot study*.* *Lasers in surgery and medicine* 48(7), 653-659 (2016).

2. Lee CC, Chen CJ, Chou CC *et al*. Lumbar Dorsal Root Ganglion Block as a Prognostic Tool Before Pulsed Radiofrequency: A Randomized, Prospective, and Comparative Study on Cost-Effectiveness*.* *World neurosurgery* 112 e157-e164 (2018).

3. Yang CL, Yang BD, Lin ML, Wang YH, Wang JL. A patient-mount navigated intervention system for spinal diseases and its clinical trial on percutaneous pulsed radiofrequency stimulation of dorsal root ganglion*.* *Spine* 35(21), E1126-1132 (2010).

4. Hsu HT, Chang SJ, Huang KF, Tai PA, Li TC, Huang CJ. Correlation between lumbar lordosis and the treatment of chronic low back pain with pulsed radiofrequency applied to the L2 dorsal root ganglion*.* *Formosan Journal of Surgery* 50(4), 125-130 (2017).

5. Tsou H-K, Chao S-C, Wang C-J *et al*. Percutaneous pulsed radiofrequency applied to the L-2 dorsal root ganglion for treatment of chronic low-back pain: 3-year experience Clinical article*.* *Journal of Neurosurgery-Spine* 12(2), 190-196 (2010).

6. Albayrak I, Apiliogullari S, Dal CN, Levendoglu F, Ozerbil OM. Efficacy of Pulsed Radiofrequency Therapy to Dorsal Root Ganglion Adding to TENS and Exercise for Persistent Pain after Total Knee Arthroplasty*.* *Journal of Knee Surgery* 30(2), 134-142 (2017).

7. Cohen SP, Sireci A, Wu CL, Larkin TM, Williams KA, Hurley RW. Pulsed radiofrequency of the dorsal root ganglia is superior to pharmacotherapy or pulsed radiofrequency of the intercostal nerves in the treatment of chronic postsurgical thoracic pain*.* *Pain Physician* 9(3), 227-235 (2006).

8. Fam BN, El-Sayed GGE, Reyad RM, Mansour I. Efficacy and safety of pulsed radiofrequency and steroid injection for intercostobrachial neuralgia in postmastectomy pain syndrome - A clinical trial*.* *Saudi journal of anaesthesia* 12(2), 227-234 (2018).

9. Kim ED, Lee YI, Park HJ. Comparison of efficacy of continuous epidural block and pulsed radiofrequency to the dorsal root ganglion for management of pain persisting beyond the acute phase of herpes zoster*.* *PloS one* 12(8), (2017).

10. Van Zundert J, Lame IE, De Louw A *et al*. Percutaneous pulsed radiofrequency treatment of the cervical dorsal root ganglion in the treatment of chronic cervical pain syndromes: A clinical audit*.* *Neuromodulation* 6(1), 6-14 (2003).

11. Zhang J, Shi D-S, Wang R. Pulsed radiofrequency of the second cervical ganglion (C2) for the treatment of cervicogenic headache*.* *Journal of Headache and Pain* 12(5), 569-571 (2011).

12. Albayrak I, Apiliogullari S, Onal O, Gungor C, Saltali A, Levendoglu F. Pulsed radiofrequency applied to the dorsal root ganglia for treatment of post-stroke complex regional pain syndrome: A case series*.* *Journal of Clinical Anesthesia* 33 192-197 (2016).

13. Apiliogullari S, Aydin BK, Onal O, Kirac Y, Celik JB. Pulsed Radiofrequency of Dorsal Root Ganglia for the Treatment of Complex Regional Pain Syndrome in an Adolescent with Poliomyelitis Sequel: A Case Report*.* *Pain Medicine* 16(7), 1369-1372 (2015).

14. Arai Y-CP, Nishihara M, Yamamoto Y *et al*. Dorsal root ganglion pulsed radiofrequency for the management of intractable vertebral metastatic pain: a case series*.* *Pain Medicine* 16(5), 1007-1012 (2015).

15. Hofmeester I, Steffens MG, Brinkert W. Chronic scrotal and inguinal pain after orchidopexy in a 13-year-old boy, treated by pulsed radiofrequency of the dorsal ganglia*.* *Journal of pediatric urology* 9(6 Pt B), e155-156 (2013).

16. Lee SY, Jang DI, Noh C, Ko YK. Successful treatment of occipital radiating headache using pulsed radiofrequency therapy*.* *Journal of Korean Neurosurgical Society* 58(1), 89-92 (2015).

17. Li J, Yin Y, Ye L, Zuo Y. Pulsed radiofrequency of C2 dorsal root ganglion under ultrasound guidance for chronic migraine: a case report*.* *Journal of pain research* 11 1915-1919 (2018).
