# Supplementary material for: Efficacy and safety of pulsed radiofrequency as a method of dorsal root ganglia stimulation for treatment of non-neuropathic pain: a systematic review
Source: BMC Anesthesiol. 2020 May 4;20:105. doi: 10.1186/s12871-020-01023-9 (PMC7199300; doi:10.1186/s12871-020-01023-9)
Supplement: Supplementary file 4 — Additional file 4: Supplementary Table 4. Individual Cochrane risk of bias judgments for randomized controlled trials. [file 12871_2020_1023_MOESM4_ESM.docx]

**Supplementary table 4. Individual Cochrane Risk of Bias assessment with explanations justifying judgment for randomized controlled trials**

| **Study** | **Random sequence generation (selection bias)** | **Allocation concealment (selection bias)** | **Blinding of participants and personnel (performance bias)** | **Blinding of outcome assessment (detection bias)** | **Incomplete outcome data (attrition bias)** | **Selective reporting (reporting bias)** | **Other bias** |
| --- | --- | --- | --- | --- | --- | --- | --- |
| **Holanda 2016 [**[**1**](#_ENREF_1)**]** | Unclear risk  It is just said that patients were randomized into three groups. Method of randomization was not reported. | Unclear risk  Not reported. | Unclear risk  All procedures were performed in same way but it is not clear from manuscript if participants were blinded. In registered protocol it is said that study was triple blinded (participants, care provider and outcome assessor). | Unclear risk  Not mentioned if outcome assessor was blinded. | Low risk  There was no lost to follow-up at one month, all participants were analyzed. | High risk  Primary and secondary outcome measures are not identical in registered protocol and published paper. For primary outcome there is no 6-month follow-up time point in manuscript. Secondary outcome published in protocol (immunochemistry analysis) is not stated and described in paper. Also Pain Relief Scale is not specified in protocol, but was reported in manuscript. | Low risk  The study appears to be free of other source of bias. |
| **Lee 2018 [**[**2**](#_ENREF_2)**]** | Unclear risk  The method of randomization is not reported; it is just said that patients were randomized. | Unclear risk  Not reported. | Unclear risk  Blinding of personnel is not mentioned while it is said that participants were unaware of sub-group to which they were assigned. | Unclear risk  Outcome measures questionnaires were administered by a nurse  who was not involved in the selection or management of patients but there were no further details about blinding of outcome assessor. | Low risk  All participants completed the study. | Unclear risk  Insufficient information to permit judgment, no data about registration of trial in Clinical Trials registry. | Low risk  The study appears to be free of other source of bias. |

**References**

[1] Holanda VM, Chavantes MC, Silva DFT, de Holanda CVM, de Oliveira JO, Wu X, et al. Photobiomodulation of the dorsal root ganglion for the treatment of low back pain: A pilot study. Lasers Surg Med. 2016;48:653-9.

[2] Lee CC, Chen CJ, Chou CC, Wang HY, Chung WY, Peng GS, et al. Lumbar Dorsal Root Ganglion Block as a Prognostic Tool Before Pulsed Radiofrequency: A Randomized, Prospective, and Comparative Study on Cost-Effectiveness. World neurosurgery. 2018;112:e157-e64.
