# Supplementary material for: Efficacy and safety of pulsed radiofrequency as a method of dorsal root ganglia stimulation for treatment of non-neuropathic pain: a systematic review
Source: BMC Anesthesiol. 2020 May 4;20:105. doi: 10.1186/s12871-020-01023-9 (PMC7199300; doi:10.1186/s12871-020-01023-9)
Supplement: Supplementary file 5 — Additional file 5: Supplementary Table 5. Individual ROBINS judgments for non-randomized studies. [file 12871_2020_1023_MOESM5_ESM.docx]

**Supplementary table 5. Individual ROBINS-I Risk of Bias assessment with explanations justifying judgment for cohort design included studies**

| **Study ID** | **Bias due to confounding** | **Bias in selection of participants into study** | **Bias in classification of interventions** | **Bias due to deviations from intended intervention** | **Bias due to missing data** | **Bias in measurement of outcomes** | **Bias in selection of the reported result** |
| --- | --- | --- | --- | --- | --- | --- | --- |
| **Albayrak 2017 [**[**1**](#_ENREF_1)**]** | **Serious**  At least one known important domain (total WOMAC, catastrophizing, depression and other comorbidities) was not appropriately measured, or not controlled for. | **Serious**  Selection into the study was not related to intervention and outcome but time of intervention and follow-up was not the same for all participants. Patients who did not complete the follow-up were excluded from the study. | **Low**  Intervention status is well defined and is based solely on information collected at the time of intervention. | **Moderate**  The important co-interventions were balanced across intervention groups. There were no deviations from the intended interventions delivered during the hospital stay (in terms of implementation or adherence) that were likely to impact on the outcome, but the adherence to at-home exercise program was not reported. | **Moderate**  There is no information about how many participants were included in each of the groups at the beginning, although all of the participants with missing data were excluded from analysis, thus unlikely to introduce bias due to the missing data. | **Serious**  The outcome measure was subjective (i.e. vulnerable to influence by knowledge of the intervention received by study participants); and the outcome was assessed by assessors aware of the intervention received by study participants. | **Moderate**  (i) The outcome measurements and analyses are clearly defined and both internally and externally consistent;  (ii) There is no indication of selection of the reported analysis from among multiple analyses;  (iii) There is no indication of selection of the cohort or subgroups for analysis and reporting on the basis of the results. |
| **Cohen 2006 [**[**2**](#_ENREF_2)**]** | **Serious**  At least one known important domain (baseline pain intensity, catastrophizing, depression and other comorbidities) was not appropriately measured, or not controlled for. | **Low**  All participants who would have been eligible for the target trial were included in the study; and participants were included into study consecutively. | **Moderate**  Both pulsed RF techniques were well defined but some aspects of the assignments of intervention status were determined retrospectively. | **Serious**  Deviations from intervention were not reported, but significantly higher proportion of participants in two PRF groups received opioids compared to the medication group. | **Low**  No losses to follow-up according to manuscript data; data were reasonably complete. | **Serious**  The outcome measure was subjective (i.e. vulnerable to influence by knowledge of the intervention received by study participants) and the outcome was assessed by assessors aware of the intervention received by study participants (no mention of blinding of outcome assessor attempted). | **Moderate**  (i) The outcome measurements and analyses are clearly defined and both internally and externally consistent;  (ii) There is no indication of selection of the reported analysis from among multiple analyses;  (iii) There is no indication of selection of the cohort or subgroups for analysis and reporting on the basis of the results; but there is no pre-registered protocol available. |
| **Kim 2017 [**[**3**](#_ENREF_3)**]** | **Serious**  Use of medication after PRF treatment was not controlled, and actual dose taken may be different than dose prescribed. It is possible that there was statistical error in the comparison of medication doses between groups. Groups were not balanced by gender and previous catheterization in the acute stage. | **Serious**  To be included in the study patients had to be present at 6 month follow-up visit. | **Moderate**  Both procedures were well defined and described in manuscript  (i) Intervention status is well defined;  and  (ii) Some aspects of the assignments of intervention status were determined retrospectively. | **Serious**  Co-interventions (use of medication after the treatment) probably were not balanced between groups. | **Moderate**  There were patients not included in the analysis because lost to follow-up, but number of patients in both groups is similar, and reasons for missing data are same in both groups. | **Serious**  Patient-reported outcomes; patients not blinded. Medical records incomplete. | **Moderate**  (i) The outcome measurements and analyses are clearly defined and both internally and externally consistent;  (ii) There is no indication of selection of the reported analysis from among multiple analyses;  (iii) There is no indication of selection of the cohort or subgroups for analysis and reporting on the basis of the results; but there is no pre-registered protocol available. |
| **Yang 2010 [**[**4**](#_ENREF_4)**]** | **Serious**  There is no data that patients were adjusted for previously taken medications or procedures as well as there is no information about taking post intervention pain medications or other therapies. | **Low**  No indications about major problems in this domain. | **Low**  Intervention is once-only surgical procedure, which is well defined. | **Moderate**  Insufficient information about potential co-interventions. | **Low**  No losses to follow-up. | **Serious**  Subjective outcome reporting by study participants who were not blinded. | **Moderate**  VAS is well defined outcome measure for pain relief. |

Acronyms: PRF = pulsed radiofrequency; RF = radiofrequency; VAS = visual analog scale; WOMAC = functional status by Western Ontario and McMaster universities osteoarthritis index.

**References**

[1] Albayrak I, Apiliogullari S, Dal CN, Levendoglu F, Ozerbil OM. Efficacy of Pulsed Radiofrequency Therapy to Dorsal Root Ganglion Adding to TENS and Exercise for Persistent Pain after Total Knee Arthroplasty. Journal of Knee Surgery. 2017;30:134-42.

[2] Cohen SP, Sireci A, Wu CL, Larkin TM, Williams KA, Hurley RW. Pulsed radiofrequency of the dorsal root ganglia is superior to pharmacotherapy or pulsed radiofrequency of the intercostal nerves in the treatment of chronic postsurgical thoracic pain. Pain Physician. 2006;9:227-35.

[3] Kim ED, Lee YI, Park HJ. Comparison of efficacy of continuous epidural block and pulsed radiofrequency to the dorsal root ganglion for management of pain persisting beyond the acute phase of herpes zoster. PLoS One. 2017;12:e0183559.

[4] Yang CL, Yang BD, Lin ML, Wang YH, Wang JL. A patient-mount navigated intervention system for spinal diseases and its clinical trial on percutaneous pulsed radiofrequency stimulation of dorsal root ganglion. Spine. 2010;35:E1126-32.
