# Supplementary material for: Efficacy and safety of pulsed radiofrequency as a method of dorsal root ganglia stimulation for treatment of non-neuropathic pain: a systematic review
Source: BMC Anesthesiol. 2020 May 4;20:105. doi: 10.1186/s12871-020-01023-9 (PMC7199300; doi:10.1186/s12871-020-01023-9)
Supplement: Supplementary file 6 — Additional file 6: Supplementary Table 6. Details about studies awaiting classification. [file 12871_2020_1023_MOESM6_ESM.docx]

**Supplementary table 6. Characteristics of randomized controlled trials awaiting assessment**

| **Trial number** | **Clinical condition** | **Comparator** | **Number of participants** | **Follow-up** | **Outcome measures** | **Status** |
| --- | --- | --- | --- | --- | --- | --- |
| NCT03204942 | Intractable metastatic pain | DRG thermal radiofrequency | 69 |  | Primary:  1. EORTC QLQ-C30 questionnaire to measure change in the intensity of pain  2. EORTC QLQ-BM22  3. VAS to evaluate pain  4. ODI  Secondary:  1. Any complications  2. Change in analgesic usage  3. Patient’s satisfaction by descriptive scale | Completed on July 30, 2018 |
| NCT03228316 | Chronic pelvic cancer pain | Superior hypogastric plexus block | 40 |  | Primary:  1. Pain intensity by VAS | Not yet recruiting |

Acronyms: DRG = dorsal root ganglion; EORTC = The European organisation for research and treatment of cancer; ODI = Oswestry disability index; QLQ-BM22 = EORTC quality of life questionnaire in patients with bone metastases; QLQ-C30 = EORTC quality of life core questionnaire; VAS = visual analog scale.
